# Lightweight Deep Learning Approaches on Edge Devices for Fetal Movement Monitoring

**DOI:** 10.3390/bios15100662

**Published:** 2025-10-02

**Authors:** Atcharawan Rattanasak, Talit Jumphoo, Kasidit Kokkhunthod, Wongsathon Pathonsuwan, Rattikan Nualsri, Sittinon Thanonklang, Pattama Tongdee, Porntip Nimkuntod, Monthippa Uthansakul, Peerapong Uthansakul

**Affiliations:** 1 School of Telecommunication Engineering, Suranaree University of Technology, Nakhon Ratchasima 30000, Thailand; d6500658@g.sut.ac.th (A.R.); rattikan.nm@gmail.com (R.N.); sittinon62@gmail.com (S.T.); mtp@sut.ac.th (M.U.); 2 Institute of Research and Development, Suranaree University of Technology, Nakhon Ratchasima 30000, Thailand; jumphoo@sut.ac.th (T.J.); kasidit.ko@sut.ac.th (K.K.); pathonsuwan.wst@sut.ac.th (W.P.); 3 School of Obstetrics and Gynecology, Institute of Medicine, Suranaree University of Technology, Nakhon Ratchasima 30000, Thailand; pattama_t@sut.ac.th; 4 School of Medicine, Institute of Medicine, Suranaree University of Technology, Nakhon Ratchasima 30000, Thailand; porntipnimk@sut.ac.th

**Keywords:** fetal movement detection, deep learning, embedded system, knowledge distillation

## Abstract

Fetal movement monitoring (FMM) is crucial for assessing fetal well-being, traditionally relying on clinical assessments or maternal perception, each with inherent limitations. This study presents a novel lightweight deep learning framework for real-time FMM on edge devices. Data were collected from 120 participants using a wearable device equipped with an inertial measurement unit, which captured both accelerometer and gyroscope data, coupled with a rigorous two-stage labeling protocol integrating maternal perception and ultrasound validation. We addressed class imbalance using virtual-rotation-based augmentation and adaptive clustering-based undersampling. The data were transformed into spectrograms using the Short-Time Fourier Transform, serving as input for deep learning models. To ensure model efficiency suitable for resource-constrained microcontrollers, we employed knowledge distillation, transferring knowledge from larger, high-performing teacher models to compact student architectures. Post-training integer quantization further optimized the models, reducing the memory footprint by 74.8%. The final optimized model achieved a sensitivity (SEN) of 90.05%, a precision (PRE) of 87.29%, and an F1-score (F1) of 88.64%. Practical energy assessments showed continuous operation capability for approximately 25 h on a single battery charge. Our approach offers a practical framework adaptable to other medical monitoring tasks on edge devices, paving the way for improved prenatal care, especially in resource-limited settings.

## 1. Introduction

Fetal movement monitoring (FMM) is a critical component of antenatal care, as fetal movements (FMs) serve as a key indicator of fetal well-being. Clinically, a significant reduction in or cessation of FMs often indicates fetal compromise and frequently precedes adverse outcomes such as stillbirth [1]. Traditional approaches to FMM rely on either periodic in-clinic assessments or maternal perception, each possessing distinct strengths and limitations. Ultrasound assessments, conducted as part of the Biophysical Profile (BPP), can clinically evaluate fetal breathing, gross body and limb movements, fetal tone, and amniotic fluid volume. However, these evaluations require specialized equipment and trained personnel, typically limiting assessments to infrequent hospital visits, thereby restricting their capability for continuous fetal behavioral monitoring. In contrast, maternal self-counting of ‘kick’ movements is a simple, cost-free method widely encouraged during late pregnancy. Despite this convenience, this subjective approach can lead to inconsistencies, as maternal perception of FM varies and may be influenced by external factors, and the burden of vigilant counting can cause anxiety [2,3]. Consequently, continuous FMM has remained undervalued despite its significant clinical importance. Continuous monitoring enables timely detection and intervention when fetal compromise occurs, significantly reducing risks of adverse outcomes, including stillbirth. However, current monitoring methods still encounter significant limitations regarding detection accuracy. False alarms may cause unnecessary stress and anxiety for expectant mothers. More critically, missed detections could lead to delayed medical intervention, placing the fetus at considerable risk. Therefore, developing consistent and accurate continuous FMM systems is essential to improve maternal–fetal safety and clinical outcomes.

Recently, advanced wearable monitoring systems have been developed specifically to overcome these issues by providing consistent monitoring of FMs. These innovative systems integrate multiple sensors, such as accelerometers, acoustic sensors, and electromyography (EMG), to provide accurate FM detection along with real-time monitoring capabilities [4,5,6]. Additionally, comprehensive wearable modules combine fetal activity monitoring with maternal vital sign assessment, enabling continuous home-based prenatal care and remote clinical evaluations without reliance on maternal perception [7,8,9]. However, while these sensor-based systems offer objective data collection, interpreting complex FM patterns and evaluating their clinical significance still require more intelligent analytical methods. Therefore, researchers have developed novel automated approaches for continuous FMM, leveraging Artificial Intelligence (AI) technology to enhance the effectiveness of fetal well-being surveillance.

Traditional AI approaches for FM classification rely primarily on classical machine learning algorithms combined with manually engineered features extracted from sensor signals such as acoustic sensors, accelerometers, electromyography (EMG), and piezoelectric sensors [10,11,12,13,14]. Algorithms like Random Forest (RF), Support Vector Machines (SVMs), k-Nearest Neighbors (k-NN), Decision Trees, AdaBoost, Multi-layer Perceptron (MLP), Logistic Regression (LR), and basic Neural Networks (NNs) have been extensively used but require substantial manual effort in feature design. For instance, Altini et al. [10,11] utilized basic statistical and variable-length features from accelerometer and EMG data, whereas Xu et al. [12] developed comprehensive statistical, morphological, and wavelet-based features. Ghosh et al. [13,14] further integrated time-domain and frequency-domain features from multiple sensors. Despite achieving satisfactory outcomes, traditional methods remain constrained by their reliance on handcrafted features, which inherently require extensive domain-specific expertise and considerable manual effort. These manually engineered features typically include statistical descriptors, wavelet-based features, and features derived from either the time domain or frequency domain. However, such features exhibit significant limitations when confronted with variations in data conditions. For instance, minor changes in sensor placement may substantially alter signal characteristics, causing previously designed features to become ineffective in capturing relevant signal patterns. Similarly, data collected from new subjects whose physiological characteristics differ from those previously encountered can lead to notable reductions in feature effectiveness. These scenarios highlight the fundamental limitation of handcrafted features in effectively adapting to variations in data conditions, consequently necessitating repeated expenditures of time and effort to redesign features whenever new or altered situations are encountered.

To address these limitations, recent studies have shifted towards deep learning techniques that automatically extract informative features directly from raw or minimally processed sensor data. Preprocessing techniques such as Power Spectral Density (PSD), Wavelet Transforms (WTs), or Short-Time Fourier Transform (STFT) transform sensor data into informative representations. Convolutional Neural Networks (CNNs) effectively capture spatial and frequency characteristics from spectrogram representations [15,16], whereas recurrent architectures like Long Short-Term Memory (LSTM) and Gated Recurrent Units (GRUs) effectively model temporal dependencies [17,18,19,20]. Although deep learning reduces manual feature engineering and improves detection accuracy, deploying these models on resource-constrained devices remains challenging due to computational and energy constraints. Cloud-centric approaches initially addressed these limitations, as demonstrated by Delay et al. [15,21], who utilized accelerometer data combined with CNNs and STFT, achieving a performance of 86–88%. However, these methods are constrained by data transmission delays and require reliable connectivity. Recent on-device AI paradigms have emerged to improve real-time practicality. Rattanasak et al. [22] achieved 88.56% performance using optimized and compressed XGBoost models on inertial measurement unit (IMU) sensor data, while Ouypornkochagorn et al. [23] reported 81.6% accuracy processing acoustic sensor data through CNNs on smartphones. Although these approaches represent important progress, there is still a significant need to develop highly efficient lightweight deep learning methods capable of direct deployment on edge devices, particularly in balancing accuracy and computational efficiency under resource constraints, which is precisely the gap this paper aims to address.

In this paper, we propose a novel lightweight deep learning framework specifically designed for real-time FMM on resource-constrained edge devices. The key contributions of our proposed framework can be summarized as follows:

- A meticulously collected dataset comprising raw six-dimensional IMU data (three-axis accelerometer and three-axis gyroscope) from 120 pregnant volunteers, with carefully validated FM events using maternal perception and simultaneous ultrasound confirmation.
- A systematic integration strategy combining knowledge distillation (KD) with INT8 post-training quantization (INT8-PTQ), explicitly optimized to significantly reduce the memory footprint and computational load for edge deployment.
- Validation of real-world deployment feasibility on a low-power ESP32-C6 microcontroller, demonstrating practical effectiveness and real-time inference capabilities independent of cloud connectivity.

This integrated approach provides a clear pathway toward widespread adoption of effective prenatal monitoring, particularly in resource-limited settings, and offers a practical framework for implementing other complex medical classification tasks onto edge computing environments.

The remainder of this paper is organized as follows. Section 2 details the materials and methods, covering the experimental design, device development, participant recruitment, and data acquisition and preprocessing, as well as the deep learning framework and evaluation strategies employed in this study. Section 3 presents the results and discussion, including baseline teacher–student performance, class-balancing strategies, effectiveness of KD, post-training quantization, comparison with existing methods, energy consumption analysis, and practical deployment feasibility. Section 4 concludes the paper by summarizing the main contributions and outlining directions for future research.

## 2. Materials and Methods

### 2.1. Experimental Design

**Figure 1:**
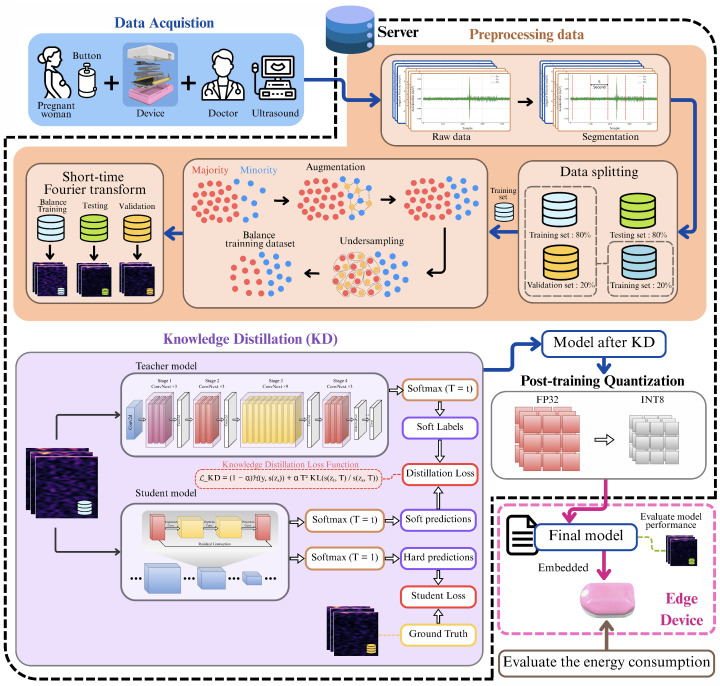
Overall framework of the proposed lightweight deep learning approach for real-time fetal movement monitoring.

The experimental design implemented in this study was structured to ensure methodological rigor, reproducibility, and validity at every stage. The approach encompasses the entire process, beginning with participant enrollment and data collection, followed by careful dataset partitioning to prevent data leakage, and a systematic preprocessing and class-balancing pipeline to extract meaningful features and address class imbalance. Finally, model development involves configuring training parameters and applying a knowledge-distillation framework to produce lightweight models suitable for deployment on resource-limited hardware. Each of these steps is described in detail below, as illustrated in Figure 1.

#### 2.1.1. Dataset Partitioning Setup

A total of 120 pregnant volunteers, between 28 and 40 gestational weeks, were recruited with institutional ethics approval. Written informed consent was obtained from all participants prior to enrollment. Each participant wore a custom-designed wearable device equipped with a six-axis IMU and a tactile event-marker button for maternal annotation.

Participants were seated comfortably in a naturalistic environment and encouraged to perform typical daily activities, such as gentle positional adjustments, minor limb movements, casual conversations, or mobile phone interactions, during the approximately 25 min recording session.

Ground-truth labeling of FM was established through a rigorous dual-confirmation protocol. Mothers initially pressed the event-marker button upon perceiving FM, which was concurrently verified by an experienced clinician performing real-time ultrasound monitoring. Only segments where maternal perception temporally coincided with ultrasound confirmation were labeled as FM events, whereas segments lacking this dual confirmation were labeled as non-fetal movements (N-FMs).

After data collection, the signals were segmented into non-overlapping 5 s windows and labeled accordingly, yielding a dataset consisting of 1073 FM and 32,770 N-FM segments. To avoid data leakage, a subject-independent hold-out approach was adopted. The dataset was partitioned such that 80% of subjects were assigned to the training group, while the remaining 20% were reserved for testing. The training subset was further divided into 80% for model training and 20% for validation, again ensuring subject independence among all subsets. After partitioning, the final dataset comprised 686 FM and 20,973 N-FM segments in the training set, 172 FM and 5243 N-FM segments in the validation set, and 215 FM and 6554 N-FM segments in the test set.

#### 2.1.2. Data Preprocessing and Class-Balancing Setup

To prepare the raw six-axis inertial data for deep learning model training and ensure that the dataset was balanced, a multi-stage preprocessing pipeline was systematically implemented. Each preprocessing step was carefully configured to extract meaningful time–frequency features, augment FM samples, and address class imbalance effectively prior to knowledge-distillation training. Importantly, class-balancing procedures were applied exclusively to the training set to preserve the integrity of the validation and testing subsets. The complete preprocessing and balancing pipeline was configured as follows:

- STFT Transformation: Raw six-axis IMU signals for each 5 s segment were transformed into time–frequency representations using the STFT. A Hamming window of 256 samples with 50% overlap was applied, and spectrograms were standardized by z-score normalization on a per-segment basis to remove inter-subject amplitude variations.
- Virtual-Rotation-Based Augmentation: Orientation invariance and FM sample expansion were achieved by randomly rotating each six-dimensional vector in 3-D space using quaternions uniformly sampled within $\pm 15^{{}^{\circ}}$ about each axis. This procedure increased FM segments from 686 to 6860 while preserving the physical relationships among axes.
- Adaptive K-Means Undersampling: To balance the dominant N-FM class, an adaptive k-means clustering method was applied with the following parameters: number of clusters k=12, maximum iterations = 300, convergence tolerance = $1\times 10^{-4}$, and a composite distance metric combining Manhattan (weight 0.7) and cosine similarity (weight 0.3). Representative N-FM samples were iteratively selected from each cluster until the FM and N-FM classes were balanced at 6860 segments each.

#### 2.1.3. Hyperparameter Setup and Model Initialization

A teacher—student KD framework was implemented to create a lightweight and accurate model suitable for microcontroller deployment. Three high-capacity teacher networks consisting of ConvNeXt-S, ResNet-101, and EfficientNet-B6 were initialized with ImageNet-pretrained weights. These models were fine-tuned to serve as knowledge sources. Three compact student networks, namely, MobileNetV2, ShuffleNet, and SqueezeNet, were trained from scratch to meet the computational constraints of the ESP32-C6 platform.

The models were trained using the Adam optimizer implemented in PyTorch v2.7.0 (Python 3.10) with an extensive grid search for key hyperparameters including learning rate, batch size, dropout rate, distillation temperature, and KD loss weight α. Early stopping was implemented based on validation loss, with training halted if no improvement was observed within 30 epochs. Each experiment was repeated five times, with results reported as mean ± standard deviation (SD).

The KD loss consisted of cross-entropy loss calculated from hard labels combined with Kullback–Leibler divergence calculated from teacher and student soft outputs. The hyperparameter search range and the optimal configuration selected for the best-performing student model are summarized in Table 1, and detailed results of the grid search are discussed comprehensively in Section 3. After training, the best-performing student network was exported into TensorFlow-Lite format and subjected to INT8-PTQ. The quantization step reduced the model size and computational complexity, enabling efficient deployment on the ESP32-C6 microcontroller platform.

### 2.2. Wearable Device Design

**Figure 2:**
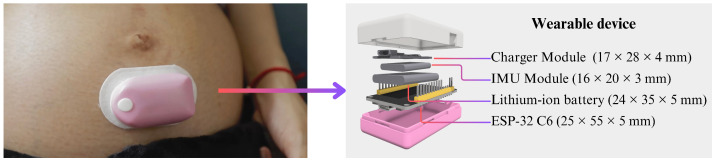
Overview of the wearable fetal movement monitoring system components.

The wearable device developed in this study was designed to be lightweight, compact, and suitable for continuous monitoring of FMs in a home environment. The core sensing element of the system was an IMU, which integrates a three-axis accelerometer and a three-axis gyroscope within a compact footprint [24]. The accelerometer supports a measurement range of ±16 g, while the gyroscope covers ±2000°/s. Both sensors operate at 16-bit resolution and can sample data at 190 Hz. An ESP32-C6 microcontroller (Espressif Systems, Shanghai, China) was used as the main processing and communication unit. This microcontroller was selected for its low power consumption, built-in wireless connectivity (Wi-Fi 6 and Bluetooth Low Energy), and compatibility with edge computing tasks. In addition to its connectivity capabilities, the ESP32-C6 features a high-performance 32-bit RISC-V core running at up to 160 MHz, along with ample SRAM and embedded 8 MB flash memory, enabling efficient real-time data processing, buffering, and local computation without relying on external resources [25]. These characteristics make the ESP32-C6 a compact yet powerful solution for our work. All electronic components were securely enclosed within a custom-designed protective casing. This enclosure was specifically engineered to offer durability, user comfort, and protection against external environmental factors such as sweat, impact, and dust. The final prototype is lightweight and unobtrusive, weighing approximately 40 g, making it suitable for attachment to the abdominal region using medical-grade adhesive patches. An overview of the device, its placement, and components is shown in Figure 2.

### 2.3. Participants

Pregnant women between 28 and 40 weeks of gestation were recruited to participate in the data collection phase at SUT Hospital. A total of 120 participants were enrolled in the study. The average age of the participants was 30.12 ± 4.85 years, with a mean gestational age at the time of data collection of 31.75 ± 2.45 weeks. The participants had an average body weight of 76.45 ± 11.90 kg, and their mean abdominal circumference was 100.32 ± 12.80 cm. Among the participants, 65 exhibited the left occiput anterior (LOA) fetal position, while the remaining 55 presented with the right occiput anterior (ROA) position.

This research was conducted with an emphasis on ethical standards. Approval was secured from the Human Research Ethics Committee of SUT (License EC-67-194, COA no. 215/2567). All participants were thoroughly informed about the study’s aims, its procedures, and their right to withdraw at any point without consequences before giving written consent. Comprehensive data protection protocols were enforced to ensure privacy and data integrity, including anonymization, secure storage, and restricted data access. Identifiable personal information was kept separate from measurement data and replaced with unique codes to maintain confidentiality. Only authorized personnel were permitted access, reinforcing the ongoing protection of participant privacy. These procedures reflect the study’s adherence to ethical principles and its commitment to safeguarding participant rights and sensitive health data.

### 2.4. Data Acquisition and Dual-Stage Labeling

Before the experimental sessions began, a medical expert provided participants with a clear explanation of the study’s objectives, duration, and data collection procedures. Participants were positioned in a comfortable and calm state. To ensure a natural and stress-free environment during data collection, they were allowed to perform light and familiar activities such as gently turning to the left or right, moving their limbs, stretching, talking, and using mobile phones.

**Figure 3:**
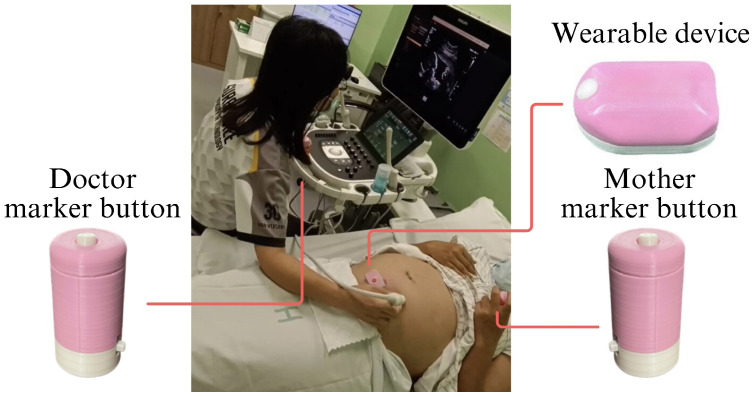
Two-stage labeling protocol for fetal movement annotation, combining maternal button press with real-time ultrasound confirmation.

The data were collected using the wearable device from 120 participants, with each recording session lasting approximately 25 min. A two-stage labeling protocol was employed to ensure accurate annotation of FM signals. First, participants were instructed to press a button whenever they perceived FM. This maternal input provided the initial label, capturing the subjective sensation of fetal motion in real time. To enhance label reliability, ultrasound imaging was simultaneously conducted during the sessions, allowing medical professionals to visually confirm FM signals. This two-stage labeling protocol served as an additional validation layer to ensure that the labeled data accurately represented true FMs, as illustrated in Figure 3.

### 2.5. Data Preprocessing

The preprocessing phase was carefully designed to ensure high-quality data for subsequent deep learning analysis. Following the methodology outlined in [12], the raw recorded signals were segmented into fixed-length windows of 5 s to maintain a consistent temporal resolution across the dataset. Each segment was then labeled as either FM or N-FM based on a two-stage labeling protocol. The N-FM class encompassed all signal activities not originating from FMs, including background noise, maternal body movements, mobile phone interactions, and other sources of interference present during data acquisition.

The resulting dataset exhibited class imbalance, consisting of 1073 FM segments and 32,770 N-FM segments. The dataset was first split into training and test sets using an 80:20 ratio, with completely separate participants. This subject-independent strategy ensured that no individual contributed data to more than one subset, thereby preventing data leakage and ensuring unbiased evaluation. This resulted in 858 FM and 26,216 N-FM segments for training, and 215 FM and 6554 N-FM segments for testing. The training set was then further divided into training and validation sets (80:20), again with non-overlapping participants, yielding 686 FM and 20,973 N-FM segments in the training set, and 172 FM and 5243 N-FM segments in the validation set. Data augmentation and balancing techniques were subsequently applied exclusively to the training subset, leaving the validation and test sets unchanged to ensure realistic evaluation scenarios.

#### 2.5.1. Virtual-Rotation-Based Data Augmentation

To address this imbalance, data augmentation was applied using the virtual rotation technique proposed by Choi et al. [26]. In this approach, the original data are transformed as if the sensor had been attached to the same body in a different orientation. This allows the model to learn motion patterns that are invariant to the sensor’s placement, enhancing its ability to generalize across varying real-world usage conditions, where sensor alignment may not be perfectly consistent. Each six-axis IMU signal, including accelerometer and gyroscope data, along with the corresponding reference orientation quaternion, is rotated virtually using a randomly generated unit quaternion.

The transformation of each sensor signal vector Sy, expressed in the original sensor coordinate system *S*, into its virtually rotated version $S^{\prime }y$ in the new coordinate system $S^{\prime }$ is defined as

(1) $$S^{\prime }y=q_{SS^{\prime }}^{-1}⊗Sy⊗q_{SS^{\prime }}$$

In this equation, Sy refers to a three-dimensional sensor measurement vector, such as linear acceleration or angular velocity, in the original coordinate frame *S*. The vector $S^{\prime }y$ represents the same measurement expressed in the rotated coordinate frame $S^{\prime }$. The unit quaternion $q_{SS^{\prime }}$ defines the rotation from frame *S* to frame $S^{\prime }$, and the rotation is applied using quaternion multiplication in a conjugation form. This formulation avoids the issue of gimbal lock, which arises in Euler angle representations when two axes become aligned and a degree of rotational freedom is lost. Quaternion multiplication preserves rotational orthogonality and enables smooth, continuous orientation changes.

To maintain consistency between the transformed sensor signals and their associated orientation labels, the reference orientation quaternion is updated as follows:

(2) $$q_{IS^{\prime }}^{\mathrm{ref}}=q_{IS}^{\mathrm{ref}}⊗q_{SS^{\prime }}$$

Here, $q_{IS}^{\mathrm{ref}}$ represents the original orientation of the sensor relative to the inertial frame *I*, and $q_{IS^{\prime }}^{\mathrm{ref}}$ is the transformed orientation relative to the new virtual frame $S^{\prime }$. By applying this transformation, the label remains aligned with the augmented sensor data.

This augmentation strategy enhances data diversity while preserving the physical consistency of sensor measurements. To address the insufficient data in the FM class, additional augmented FM segments were generated and incorporated into the training set. This method not only expands the dataset but also enhances the model’s ability to handle variations in sensor orientation, which is particularly important in wearable devices, where sensor placement may differ between users or across usage sessions.

Although virtual rotation is a physically grounded method for augmenting IMU signals, generating synthetic samples must be approached with caution, particularly in the context of medical data. Unlike generic datasets, medical signals often contain subtle patterns and clinically significant variations that are difficult to replicate artificially. Therefore, augmentation should be applied with care to avoid potential adverse effects. To further investigate this issue, we conducted experiments, presented in Section 3.2, where model performance was systematically evaluated under different degrees of augmentation.

After the augmentation step, the training dataset comprised 6860 FM segments and 20,973 N-FM segments, reflecting an improvement but still exhibiting imbalance. This imbalance was further addressed through adaptive clustering-based undersampling, as described in the following section.

#### 2.5.2. Adaptive K-Means Clustering-Based Undersampling

Although data augmentation improved the class distribution, a residual imbalance remained. To address this, we applied an adaptive k-means clustering-based undersampling method to the majority N-FM class in the training set, following the approach of Zhou et al. [27]. This technique dynamically estimates the optimal number of clusters *k* based on dataset characteristics and ensures a stratified representation of the majority samples. The clustering minimizes the within-cluster sum of squared errors (WCSS):

(3) $$J=\sum_{j=1}^{k}\sum_{i=1}^{n_{j}}{∥x_{i}^{\left(j\right)}-\mu_{j}∥}^{2}$$

where *k* denotes the number of clusters, $n_{j}$ is the number of instances in cluster *j*, $x_{i}^{\left(j\right)}$ is the *i*-th sample in cluster *j*, and $\mu_{j}$ is the cluster centroid.

Post-clustering, representative samples are selected from each cluster in proportion to its size using stratified sampling. Two selection criteria are employed: the Manhattan distance for numerical features to ensure centrally representative samples, and cosine similarity for directional features to maintain sample diversity. This dual-perspective sampling ensures that the selected instances are central as well as diverse, reducing redundancy while maintaining the structural integrity of the majority class. The final sample set is balanced to match the number of instances in the minority FM class.

As a result, after applying both virtual-rotation-based data augmentation and adaptive k-means clustering-based undersampling, a balanced training dataset was obtained, comprising an equal number of FM and N-FM classes, specifically 6860 FM segments and 6860 N-FM segments.

#### 2.5.3. Short-Time Fourier Transform

To transform raw inertial signals into time–frequency representations suitable for deep learning, each trial segment was first processed by combining data from all six IMU channels into three channels, merging signals from each axis pair. These combined channels were then processed using the STFT [28,29], defined in Equation (4). Prior to transformation, z-score normalization was applied on a per-trial basis to standardize the signal distribution and mitigate inter-trial variability.

(4) $$X\left(\tau ,f\right)=\int_{-\infty }^{\infty }x\left(t\right)w\left(t-\tau \right)e^{-j2\pi ft}\;dt$$

where x(t) denotes the input signal, w(t−τ) is a windowing function centered at time τ, *t* represents time, and *f* corresponds to frequency. To balance time and frequency resolution, a fixed window length with 50% overlap between adjacent windows was applied. Following the STFT, the resulting spectrograms were converted to a logarithmic scale to compress the dynamic range and enhance feature contrast. To ensure consistency across datasets, global normalization was performed using computed statistics.

### 2.6. Knowledge Distillation

KD is a model compression technique utilized to enable the deployment of FM detection models on resource-constrained edge devices. KD facilitates the transfer of knowledge from a computationally intensive teacher model to a lightweight student model, enabling the student to approximate the teacher’s performance with significantly reduced memory usage and inference latency. This method is particularly advantageous for real-time applications under strict power and computational constraints, such as embedded systems and edge computing platforms. The concept of KD was first introduced by Hinton et al. [30]. KD leverages the soft target distributions produced by the teacher model. These soft targets, derived by applying temperature scaling to the teacher’s logits, provide rich inter-class relationship information that is typically absent when using conventional hard (one-hot) labels. Training the student model with these soft targets results in improved generalization and more nuanced decision-making capabilities.

The KD training objective combines two loss components: the cross-entropy loss $H(y,s\left(z_{s}\right))$ computed between the student’s predictions and the true labels (hard targets), and the Kullback–Leibler (KL) divergence $\mathrm{KL}(s\left(z_{t},T\right)∥s\left(z_{s},T\right))$ between the softened output distributions of the teacher and student models. The combined loss function is expressed as

(5) $$L_{\mathrm{KD}}=\left(1-\alpha \right)H\left(y,s\left(z_{s}\right)\right)+\alpha T^{2}\mathrm{KL}\left(s\left(z_{t},T\right)∥s\left(z_{s},T\right)\right)$$

where α is a weighting factor that balances the contributions of hard and soft targets, $z_{t}$ and $z_{s}$ represent logits from the teacher and student models, respectively, *T* denotes the temperature parameter for scaling, and s(·,T) denotes the softmax function with temperature scaling.

#### 2.6.1. Teacher Models

Three state-of-the-art neural architectures were selected as teacher models based on their superior representational capabilities and empirical performance on visual and sensor-based classification tasks:

- ConvNeXt-S: A modern convolutional architecture incorporating transformer-inspired design elements (e.g., LayerNorm, GELU activation, and depthwise convolutions) while maintaining computational efficiency. It offers hierarchical feature extraction and strong generalization capabilities [31].
- ResNet-101: A deep residual network characterized by identity skip connections, which stabilize training and alleviate the vanishing gradient problem. Its substantial depth facilitates robust extraction of high-level spatial–temporal features [32].
- EfficientNet-B6: A compound-scaled architecture designed by systematically balancing depth, width, and resolution. Incorporating Mobile Inverted Bottleneck Convolution (MBConv) and Squeeze-and-Excitation (SE) blocks, it achieves high accuracy with fewer parameters, providing computational efficiency and robust multi-scale feature representations [33].

These teacher models collectively offer a diverse range of architectural strengths, from deep residual learning and compound scaling to transformer-inspired convolutional design. Their high representational power and proven performance on both visual and sensor-based tasks make them well-suited for guiding compact student networks through KD. We initialized each teacher model with ImageNet-pretrained weights and fine-tuned them on our dataset to adapt to the characteristics of the task.

#### 2.6.2. Student Models

To satisfy the memory and compute constraints of the ESP32 microcontroller, three compact neural architectures were selected as student models:

- MobileNetV2: Employs depthwise separable convolutions combined with inverted residual blocks and linear bottlenecks. This design significantly improves computational efficiency and facilitates efficient information flow, enabling effective feature representation using minimal parameters [34].
- ShuffleNet: Utilizes pointwise group convolutions and channel shuffling operations to enhance feature diversity and reduce computational overhead. Its architectural approach effectively promotes inter-channel interactions and structured, efficient feature extraction [35].
- SqueezeNet: Features a highly compact architecture employing Fire modules, which consist of alternating 1 × 1 and 3 × 3 convolutions. The approach achieves substantial parameter reduction and aggressive model compression while maintaining strong representational capabilities and accuracy [36].

These student architectures were selected due to their advantageous balance between performance and resource efficiency, making them particularly suitable for deployment on resource-constrained embedded systems. To align with the deployment requirements, each model was initialized with random weights and trained from scratch on our dataset. Additionally, to ensure full compatibility with the TensorFlow Lite Micro (TFLM) inference engine and the hardware limitations of the ESP32-C6, each student model was further customized through structural simplification and layer-wise modification. These adaptations ensure that all models can be successfully compiled, quantized, and executed within the strict memory and compute budgets of the target platform.

After identifying the best-performing student model, we subjected it to INT8-PTQ to further investigate and maximize deployment efficiency. Details of this compression method are presented in the following section.

### 2.7. Post-Training Quantization

Quantization is a crucial optimization technique for deploying deep learning models on resource-constrained embedded systems [37]. In this study, we adopted INT8-PTQ as the main compression strategy. This approach minimizes model size and computational load, enabling efficient inference on the ESP32-C6 microcontroller.

Full integer quantization converts all model components, including weights, activations, inputs, and outputs, into 8-bit integer format. This fixed-point representation allows the model to run using efficient integer arithmetic operations, which match the capabilities of embedded AI hardware. This strategy significantly reduced both the model’s size and memory usage, while maintaining sufficient classification performance for real-time FM detection [38]. The detailed evaluation of INT8-PTQ in terms of size reduction and classification performance is presented in the following sections.

### 2.8. Model Performance Evaluation Criteria

To evaluate the model’s performance, the metrics considered in this study include SEN, PRE, and F1, which are defined as follows:

(6) $$\mathrm{Sensitivity}=\frac{\mathrm{TP}}{\mathrm{TP}+\mathrm{FN}}$$

(7) $$\mathrm{Precision}=\frac{\mathrm{TP}}{\mathrm{TP}+\mathrm{FP}}$$

(8) $$F1-\mathrm{score}=2\times \frac{\mathrm{Precision}\times \mathrm{Sensitivity}}{\mathrm{Precision}+\mathrm{Sensitivity}}$$

Here, a true positive (TP) indicates that the model correctly detects FM when the fetus is actually moving. A true negative (TN) indicates that the model correctly identifies no movement when the fetus is not moving. A false positive (FP) refers to a case where the model incorrectly detects movement when there is none, whereas a false negative (FN) refers to a failure to detect movement when the fetus is actually moving.

### 2.9. Clinical Interpretation of Performance Metrics and Risk Considerations

SEN, PRE, and F1 are key metrics for evaluating the reliability of FMM systems. SEN reflects the model’s ability to detect movements that truly occur. For example, when the fetus moves, a system with high SEN will successfully capture almost all of these events without missing them. PRE, on the other hand, indicates the proportion of alerts that are correct. A high PRE means that when the system signals an FM event, it is very likely to represent a real occurrence rather than noise or maternal activity. F1 integrates both SEN and PRE, providing a balanced measure of how well the system avoids missed detections while also minimizing false alarms. This balance is crucial for meaningful interpretation in a clinical context.

Moreover, these metrics highlight two important types of clinical risk. An FN occurs when the fetus actually moves but the system fails to detect it. This may cause unnecessary concern for the mother, as she may believe that the fetus is not moving. An FP occurs when the system signals a movement even though none has occurred, which may create false reassurance by overestimating movement counts when the fetus is in fact moving less. Both error types can negatively affect clinical decision-making and maternal well-being. Therefore, minimizing both FN and FP is essential to ensuring that FMM systems remain safe, reliable, and clinically useful.

## 3. Results and Discussion

This section presents a comprehensive evaluation of our proposed lightweight deep learning framework for real-time FM detection on embedded edge devices. To ensure consistent performance, all training procedures were conducted using early stopping mechanisms, in which training was halted when the validation loss failed to improve over a defined number of epochs, thereby reducing the risk of overfitting. Hyperparameters were optimized through an exhaustive grid search, which explored learning rates of 0.0001,0.001, batch sizes of 16,32,64, and dropout rates of 0.2,0.3,0.5. F1 was selected as the primary performance metric due to its ability to balance sensitivity and precision. To further ensure consistency and reproducibility, each experiment was repeated five times, and the results are reported as mean values with SDs.

The results are structured into seven main parts. First, we establish baseline performance by comparing high-capacity teacher models with compact student networks under imbalanced data conditions. Second, we investigate how class-balancing strategies enhance the detection of minority class instances, aiming to address performance degradation caused by skewed label distributions. Third, we evaluate the effectiveness of KD in transferring classification capability from teacher to student models, enabling lightweight models to retain predictive strength. Fourth, we examine INT8-PTQ as a model compression technique to significantly reduce memory footprint while maintaining inference accuracy suitable for embedded deployment. Fifth, we compare the performance of our approach against existing methods from the literature. Sixth, we analyze energy consumption during real-time inference on the target device to assess runtime efficiency. Finally, we explore the practical feasibility and complexity of deploying the proposed framework in wearable monitoring applications, focusing on implementation simplicity, responsiveness, and user usability.

### 3.1. Evaluation of Teacher and Student Models on Imbalanced Data

This experiment evaluates the baseline performance of teacher and student models trained on a highly imbalanced dataset without any balancing techniques. The objective is to examine the behavior of each architecture when the minority class is severely underrepresented in the training data.

As shown in Table 2, all models demonstrate poor performance across SEN, PRE, and F1. This is a common effect of severe class imbalance, where the training process tends to prioritize the dominant class in order to minimize the overall loss [39]. Since standard cross-entropy loss treats each sample equally, the models become biased toward the majority class and fail to adequately learn decision boundaries for the minority class.

These findings highlight the significant impact of class imbalance on model performance, regardless of architectural scale. Both teacher and student models demonstrated limited ability to accurately detect the minority class when trained on unbalanced data. To address this limitation, the subsequent experiments incorporate class-balancing strategies during training. These strategies aim to improve the model’s performance by alleviating the bias toward the majority class.

### 3.2. Performance of Teacher and Student Models with Class Balancing

Following the initial evaluation on imbalanced data, this experiment explores the impact of class-balancing strategies on the performance of teacher and student models. First, we applied only undersampling to the majority class. Second, we applied a combination of both augmentation and undersampling. Third, we applied only augmentation to the minority class. The goal is to determine how different balancing strategies influence the learning dynamics of each architecture and to identify the optimal configuration for maximizing minority class detection.

Table 3 presents the F1 results across all models under different augmentation and undersampling ratios. As observed, the application of any balancing strategy yields clear improvements over the imbalanced baseline. However, when examined individually, undersampling and augmentation are less effective than their combined use. Undersampling alone substantially reduces the number of training samples, limiting the model’s ability to generalize, whereas augmentation alone often generates redundant synthetic samples that contribute little new information. In contrast, combining both strategies provides complementary benefits: undersampling mitigates class dominance, while augmentation enriches data diversity. The best results were achieved when augmentation was applied at ×10 and undersampling at ×3.06, producing the highest overall F1 across both teacher and student models. These findings underscore the importance of applying class balancing with carefully chosen proportions, as an optimal ratio not only alleviates bias but also ensures sufficient data volume and diversity for effective model learning.

To better understand the performance at the optimal balance point, Table 4 summarizes the SEN, PRE, and F1 of each model in the best-performing run. The results confirm that both teacher and student models benefit from balanced training, with significant improvements in all key metrics.

Among the teacher models, ConvNeXt (T1) achieved the highest overall performance. This strong performance is attributed to its architectural enhancements, including large kernel convolutions, depthwise separable convolutions, and LayerNorm-based normalization, which are inspired by the transformer design [40]. These features enable ConvNeXt to capture both fine-grained local patterns and broader contextual information from the time–frequency spectrograms derived from FM signals. EfficientNet-B6 (T3) and ResNet-101 (T2) also performed well, highlighting the importance of deep, well-structured networks in handling complex biomedical time-series data.

In contrast, the student models, designed for deployment on resource-constrained edge devices, showed a clear trade-off between model complexity and classification performance. Custom-MobileNetV2 (S1) achieved the highest F1 at 87.21 ± 0.05%, followed by Custom-ShuffleNet (S2) and Custom-SqueezeNet (S3) with 84.27 ± 0.06% and 81.30 ± 0.05%, respectively. The lower performance can be attributed to architectural limitations, including reduced depth, fewer parameters, and narrower receptive fields, which hinder the ability to capture subtle spatiotemporal features in FM signals. Although such model compression is necessary to meet the resource constraints of embedded deployment, the resulting drop in classification performance may limit their suitability for clinical applications, where high reliability and diagnostic confidence are essential [41].

All these results highlight the necessity of improving student model performance while keeping the model size small. To address this, we adopt KD to transfer classification capabilities from high-performing teacher models to compact student models without increasing their parameter count [42,43,44]. The best-performing teacher and student models were selected as the basis for the distillation process in the next stage.

### 3.3. Evaluation of Knowledge Distillation

**Figure 4:**
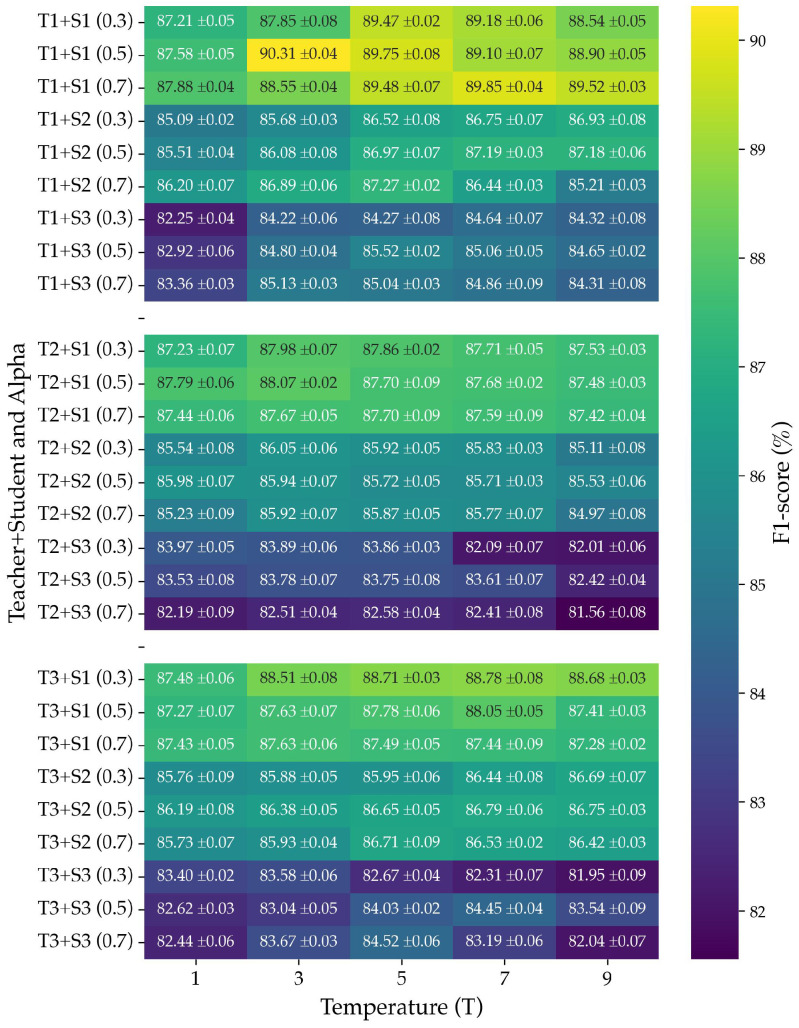
Effect of temperature (*T*) and alpha (α) on knowledge distillation performance across teacher–student model pairs.

To improve the performance of lightweight student models without increasing their parameter size, we employed a KD framework in which the student network is trained to mimic the behavior of a larger teacher model. A grid search was conducted to identify optimal distillation parameters by systematically exploring different teacher–student combinations, temperature values (*T*), and α settings. Figure 4 presents the F1 obtained from all nine teacher–student pairs under various configurations.

The results indicate that both α and *T* significantly impact distillation performance. For most teacher–student combinations, moderate values of α=0.5 consistently outperformed both lower (α=0.3) and higher (α=0.7) settings. Specifically, the best results were achieved by student S1 (T1 + S1) at α of 0.5 and T=3 (F1 = 90.31 ± 0.04%), student S2 (T1 + S2) at α of 0.7 and T=5 (F1 = 87.27 ± 0.02%), and student S3 (T1 + S3) at α of 0.5 and T=5 (F1 = 85.52 ± 0.02%). This suggests that an appropriate balance between supervision from ground-truth labels and guidance from softened teacher outputs is critical for effective knowledge transfer. Similarly, temperature values in the moderate range (T=3 to T=5) yielded the highest F1 across nearly all configurations, whereas extreme temperatures such as T=1 or T=9 typically degraded performance. Lower temperatures produce sharper distributions that offer limited inter-class information, whereas higher temperatures overly smooth outputs, obscuring class-specific distinctions.

In the subsequent analysis, each student’s best-performing configuration will be further evaluated through a comprehensive comparison of classification performance (F1), model complexity (number of parameters), and computational cost (FLOPs) to identify the most cost-effective student model for practical deployment.

**Figure 5:**
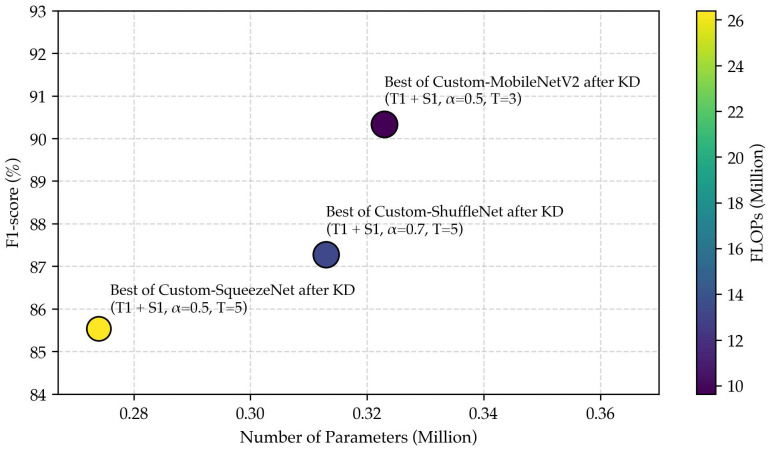
Trade-off between performance, model size, and FLOPs in knowledge-distilled student models.

Based on the analysis presented in Figure 5, the T1 + S1 combination achieves the highest F1, clearly outperforming T1 + S2 and T1 + S3. Moreover, T1 + S1 has a relatively low computational cost, which is significantly lower than others. Despite having slightly more parameters compared to T1 + S2 and T1 + S3, the enhanced predictive performance and reduced computational load make T1 + S1 the most favorable model overall. Therefore, based on these results, the T1 + S1 model was selected for the subsequent quantization stage.

### 3.4. Model Compression

To meet the deployment requirements of our FMM system on the ESP32-C6, we applied INT8-PTQ to the best-performing student model. After INT8-PTQ, the model size was reduced from 1.23 MB to 0.31 MB, representing a 74.8% reduction. This compression allowed the model to fit comfortably within the ESP32-C6 while still maintaining real-time processing capabilities.

The final quantized model achieved a SEN of 90.05 ± 1.60%, PRE of 87.29 ± 1.54%, and F1 of 88.64 ± 1.56%, representing only a 1.67 percentage point reduction compared to the pre-INT8-PTQ model. This minimal degradation indicates that the essential predictive characteristics of the model were well preserved despite the reduction in numerical PRE. From a deployment standpoint, the INT8 model executed reliably and efficiently on the ESP32-C6 using the TFLM runtime. Throughout real-time inference, the system exhibited stable behavior, with no memory overflow or runtime errors observed.

From a clinical perspective, the reported SEN of 90.05% reflects the model’s strong ability to detect FM events, which is critical for avoiding missed detections that could delay medical intervention. Meanwhile, the PRE of 87.29% suggests that most detected events are TP, thereby reducing unnecessary false alarms that could cause anxiety for expectant mothers. The observed SEN and PRE indicate that it offers a useful approach to supporting FMM, particularly in contexts where continuous clinical oversight is limited.

In conclusion, INT8-PTQ was instrumental in enabling the final model to meet both memory and performance constraints. It allowed the proposed system to operate fully on-device, supporting real-time FM detection in a form factor suitable for continuous home monitoring, particularly in resource-limited settings.

### 3.5. Comparison with Existing Methods

The comparative analysis in Table 5 shows the progression of methods for FMM. Early studies such as Altini et al. [10,11] employed basic statistical features and achieved only modest SEN. Xu et al. [12] expanded this approach with a richer set of handcrafted descriptors, improving PRE and F1 but still requiring intensive feature engineering. Ghosh et al. [13] shifted attention toward time–frequency representations, which helped capture spectral information but delivered only moderate performance. More recently, Rattanasak et al. [22] advanced feature-based machine learning with optimized statistical descriptors, achieving strong results (F1 = 88.56%) and setting a competitive benchmark among traditional approaches.

In contrast, this work employs a deep learning framework that directly processes STFT spectrograms. Before compression, the model achieved an F1 of 90.31%, exceeding all baselines, and retained an F1 of 88.64% after quantization. Although this result appears numerically close to that of Rattanasak et al. [22] the advantages of deep learning remain clear. Feature-based methods are often vulnerable to variations in sensor placement and physiological differences between subjects, necessitating repeated redesign and optimization. By learning discriminative patterns directly from raw spectrograms, the proposed approach maintains consistent performance without manual adjustments, underscoring the long-term value of deep learning even when compressed models report performance metrics similar to handcrafted approaches.

### 3.6. Energy Consumption During Real-Time Inference

Energy efficiency is a critical consideration for wearable systems, particularly in scenarios requiring continuous, real-time monitoring. To assess the practical feasibility of our proposed system under such constraints, we conducted an evaluation of the power consumption associated with on-device inference on the ESP32-C6 microcontroller. Specifically, we employed a Keysight InfiniiVision DSOX2002A oscilloscope (Keysight Technologies, Santa Rosa, CA, USA) to measure the system’s power usage during real-time operation.

**Figure 6:**
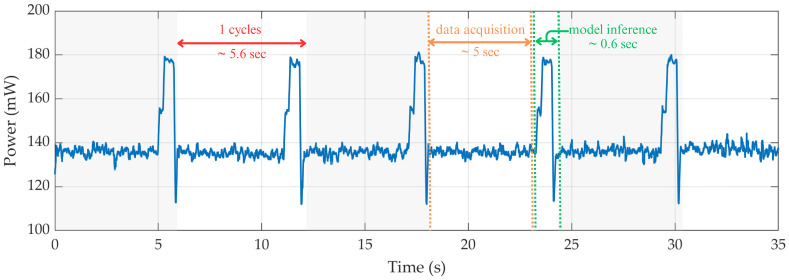
Power consumption profile of ESP32-C6 during real-time inference cycles. The grey shaded areas represent one complete operation cycle (~5.6 s), consisting of approximately 5 s of data acquisition followed by ~0.6 s of model inference.

Figure 6 presents the power profile across five consecutive inference cycles. Each cycle consisted of data acquisition, on-device preprocessing, and quantized model inference. During idle operation, the system maintained a baseline power consumption of approximately 135 mW. Each full inference cycle involved a 5 s data acquisition phase, followed by processing and inference lasting approximately 0.63 s, which also reflects the system’s per-inference latency. The active phase generated a brief power spike, reaching a peak of 179.8 mW, before returning to the baseline level.

To validate the system’s suitability for continuous wearable use, we conducted battery life evaluations using multiple lithium–polymer batteries, all rated at 3.7 V. As shown in Table 6, the operating time increases proportionally with battery capacity, ranging from 6 h with a 250 mAh battery to 50 h with a 2000 mAh unit. These tests were conducted under the same periodic inference and idle conditions characterized in the measured power profile. The results confirm that the system maintains high energy efficiency across various power configurations and demonstrates practical viability for real-time monitoring in low-power, battery-operated wearable applications.

However, while larger batteries offer significantly longer operating times, they also increase the overall size and weight of the device. This trade-off between battery capacity and physical form factor should be carefully considered in future hardware designs, especially in applications requiring long-term wearable comfort and portability.

### 3.7. Practical Feasibility and Complexity of Real-Time Deployment

The feasibility of real-time deployment was carefully considered from multiple perspectives, including hardware constraints, energy consumption, and usability in wearable devices. The integration of KD and post-training quantization produced a compact model capable of running on resource-limited edge microcontrollers without the need for external memory or continuous cloud connectivity. The framework also demonstrated the ability to process data segments within the required real-time constraints, ensuring that incoming signals can be analyzed promptly while leaving sufficient idle time for energy savings. In the context of wearable devices, this translates into continuous monitoring capability over extended periods without frequent recharging, which is essential for patient compliance and practical usability.

The complexity of the proposed framework should be considered as two stages, development and deployment. During development, data preprocessing itself introduces nontrivial complexity, particularly in addressing class imbalance. The balancing strategy required the integration of augmentation techniques, such as virtual rotation of FM signals, combined with undersampling of non-movement segments. These steps, while effective in mitigating data skew, added design considerations and validation to ensure that the augmented signals preserved physiological plausibility. Following this stage, KD requires careful pairing of teacher and student models as well as fine-tuning of distillation hyperparameters to effectively transfer knowledge without underfitting or overfitting. Quantization further requires calibration to align model weights and activations with integer representations. It also requires validation to ensure that diagnostic accuracy is not significantly compromised. However, once the model has been optimized and compressed, the deployment complexity is drastically reduced. The final quantized student model can be executed as a fixed lightweight model, relying only on simple integer arithmetic fully supported by the hardware. As a result, the runtime system remains efficient and easy to maintain, requiring no further tuning or adaptation by the end user.

Nevertheless, it is important to note that the dataset used in this study was collected under controlled conditions and did not include diverse real-world maternal activities such as walking, exercising, or daily routines. This limitation may affect the generalizability of the proposed framework, because models trained on relatively static and controlled data may not fully capture the variability and noise introduced by dynamic maternal activities. As a result, model performance may vary in less constrained environments, where additional motion artifacts and background activity signals are present.

## 4. Conclusions and Future Work

This study presented a comprehensive and energy-efficient deep learning framework for real-time FMM on embedded edge devices. By leveraging a combination of virtual-rotation-based data augmentation, adaptive clustering-based undersampling, and STFT, we constructed a balanced and discriminative dataset from raw six-axis IMU signals. A KD framework was then employed to transfer the representational power of high-capacity teacher models to compact student networks, which were subsequently optimized through INT8-PTQ. The final lightweight model achieved strong performance while drastically reducing memory and computational demands, enabling reliable on-device inference on the ESP32-C6 microcontroller. Furthermore, the system demonstrated high energy efficiency, with battery evaluations confirming its feasibility for long-term, low-power wearable use in real-world contexts.

Beyond technical efficiency, the proposed approach contributes a practical design blueprint for adapting other complex biomedical AI tasks to resource-constrained environments, where real-time inference, usability, and patient compliance are critical. Importantly, the study also highlighted clinical considerations, showing that high SEN and PRE are necessary to minimize FN and FP, both of which carry direct implications for maternal well-being and clinical decision-making.

Future work will focus on expanding data collection to include dynamic maternal activities such as walking, exercise, routine daily behaviors, and diverse real-world scenarios in order to enhance the applicability and reduce the risks of FN and FP. In addition, we plan to expand recruitment across multiple clinical sites to enable independent external validation and LOSO-CV for more rigorous evaluation. Furthermore, large-scale clinical validation in real-world home monitoring settings will be pursued to comprehensively assess long-term reliability, usability, and impacts on maternal–fetal health outcomes.

## Figures and Tables

**Table 1:** Hyperparameter search space for model training.

Parameter	Search Range
Learning rate	{0.0001, 0.001}
Batch size	{16, 32, 64}
Dropout rate	{0.2, 0.3, 0.5}
Distillation temperature	{1, 3, 5, 7, 9}
Alpha (KD loss weight)	{0.3, 0.5, 0.7}
Epochs (max)	300

**Table 2:** Performance of teacher and student models under class imbalance.

Method	Sensitivity (%)	Precision (%)	F1-Score (%)	Params (M)
Teacher				
T1: ConvNeXt	27.21 ± 0.96	32.27 ± 1.68	29.52 ± 1.17	49.46
T2: ResNet-101	24.42 ± 0.82	27.53 ± 1.05	25.88 ± 0.86	42.51
T3: EfficientNet-B6	23.72 ± 1.12	26.64 ± 0.61	25.08 ± 0.67	43.23
Student				
S1: Custom-MobileNetV2	22.44 ± 1.34	24.63 ± 0.60	23.48 ± 1.00	0.32
S2: Custom-ShuffleNet	20.70 ± 0.78	22.08 ± 0.52	21.36 ± 0.65	0.31
S3: Custom-SqueezeNet	18.72 ± 1.39	19.59 ± 1.52	19.15 ± 1.45	0.27

**Table 3:** Performance of teacher–student models under different augmentation and undersampling ratios.

Run	Aug (FM)	Under (N-FM)	F1 (%)					
			**T1**	**T2**	**T3**	**S1**	**S2**	**S3**
R1	None (686)	×30.57 (686)	82.03 ± 0.09	79.50 ± 0.12	80.06 ± 0.12	75.02 ± 0.10	72.51 ± 0.04	69.93 ± 0.01
R2	×3 (2058)	×10.19 (2058)	83.91 ± 0.10	81.33 ± 0.11	81.91 ± 0.09	76.77 ± 0.07	74.16 ± 0.05	71.53 ± 0.03
R3	×5 (3430)	×6.11 (3430)	88.69 ± 0.05	85.99 ± 0.07	86.54 ± 0.06	81.12 ± 0.12	78.42 ± 0.08	75.63 ± 0.07
R4	×7 (4802)	×4.37 (4802)	92.05 ± 0.04	89.20 ± 0.04	89.80 ± 0.12	84.15 ± 0.09	81.35 ± 0.07	78.43 ± 0.07
R5	×9 (6174)	×3.39 (6174)	94.91 ± 0.02	91.97 ± 0.02	92.61 ± 0.03	86.76 ± 0.05	83.88 ± 0.12	80.91 ± 0.14
R6	×10 (6860)	×3.06 (6860)	95.35 ± 0.03	92.45 ± 0.03	93.06 ± 0.02	87.21 ± 0.05	84.27 ± 0.06	81.30 ± 0.05
R7	×11 (7546)	×2.85 (7546)	94.97 ± 0.02	92.06 ± 0.03	92.75 ± 0.03	86.87 ± 0.07	84.02 ± 0.06	80.95 ± 0.10
R8	×12 (8232)	×2.55 (8232)	94.92 ± 0.03	91.95 ± 0.03	92.68 ± 0.04	86.77 ± 0.00	83.96 ± 0.13	80.88 ± 0.12
R9	×15 (10,290)	×2.04 (10,290)	94.62 ± 0.03	91.69 ± 0.05	92.34 ± 0.05	86.52 ± 0.06	83.62 ± 0.07	80.65 ± 0.10
R10	×20 (13,720)	×1.53 (13,720)	93.95 ± 0.04	91.05 ± 0.04	91.67 ± 0.05	85.89 ± 0.12	83.05 ± 0.14	80.06 ± 0.05
R11	×25 (17,150)	×1.22 (17,150)	93.47 ± 0.02	90.59 ± 0.06	91.20 ± 0.05	85.48 ± 0.11	82.59 ± 0.09	79.65 ± 0.09
R12	×30.57 (20,973)	None (20,973)	93.01 ± 0.04	90.15 ± 0.03	90.73 ± 0.05	85.02 ± 0.07	81.94 ± 0.40	79.29 ± 0.10

**Table 4:** Performance of best configuration teacher and student models.

Method	Sensitivity (%)	Precision (%)	F1-Score (%)	Params (M)
Teacher				
T1: ConvNeXt	95.34 ± 0.41	95.35 ± 0.37	95.35 ± 0.03	49.46
T2: ResNet-101	92.47 ± 0.41	92.42 ± 0.34	92.45 ± 0.03	42.51
T3: EfficientNet-B6	93.60 ± 0.39	92.53 ± 0.32	93.06 ± 0.02	43.23
Student				
S1: Custom-MobileNetV2	86.19 ± 0.41	88.23 ± 0.31	87.21 ± 0.05	0.32
S2: Custom-ShuffleNet	82.56 ± 0.42	86.06 ± 0.29	84.27 ± 0.06	0.31
S3: Custom-SqueezeNet	81.39 ± 0.26	81.20 ± 0.15	81.30 ± 0.05	0.27

**Table 5:** Comparative performance of existing and proposed methods for fetal movement detection.

Reference	Feature Extraction	Classifier	Performance
Altini et al. (2016) [10]	Mean, SD, interquartile range, correlation between axes and reference sensor	ML (Random Forest)	SEN = 75.00%
Altini et al. (2017) [11]	Mean, SD, interquartile range, min, max, sum, magnitude, axis correlation	ML (Random Forest)	SEN = 74.00%
Xu et al. (2022) [12]	Min, max, mean, median, SD, entropy, kurtosis, area metrics, morphological features, Haar DWT	ML (Extra Trees)	SEN = 82.40%, PRE = 86.10%, F1 = 84.20%
Ghosh et al. (2024) [13]	Spectrogram + Welch PSD	ML (Neural Network)	SEN = 82.00%, PRE = 76.00%, F1 = 79.00%
Rattanasak et al. (2025) [22]	Max, Min, Mean, Median, Mode, SD, Variance, Coefficient of Variation, Skewness, Kurtosis	ML (XGBoost)	SEN = 90.00%, PRE = 87.46%, F1 = 88.56%
This Work (2025)	STFT	DL (MobileNetV2)	Before compression SEN = 94.88%, PRE = 86.17%, F1 = 90.31%
			After compression SEN = 90.05%, PRE = 87.29%, F1 = 88.64%

**Table 6:** Comparison of operating time and weight at different battery capacities.

Battery Capacity (mAh)	Operating Time (h)	Battery Weight (g)
250	6	6.6
650	16	10.6
1000	25	17.2
1650	41	28.4
2000	50	34.4

## Data Availability

The dataset is available from the authors upon reasonable request.

## References

[1] Smith V., Muldoon K., Brady V., Delaney H. (2021). Assessing Fetal Movements in Pregnancy: A Qualitative Evidence Synthesis of Women’s Views, Perspectives and Experiences. BMC Pregnancy Childbirth.

[2] Mangesi L., Hofmeyr G.J., Smith V., Smyth R.M. (2015). Fetal Movement Counting for Assessment of Fetal Wellbeing. Cochrane Database Syst. Rev..

[3] El-Sayed H.M.E.-S., Hassan S.I., Aboud S.A.H.H., Al-Wehedy A.I. (2018). Effect of Women Self Monitoring of Fetal Kicks on Enhancing Their General Health Status. Am. J. Nurs. Res..

[4] Ahmad S.G., Arif M.A., Hassan A., Ayyub K., Munir E.U., Ramzan N. (2025). IoT-Based Smart Wearable Belt for Tracking Fetal Kicks and Movements in Expectant Mothers. IEEE Sens. J..

[5] Pratheesha D., Shashi Raj K., Yadav S., Bharadvaj S. Fetal Heart Rate and Kicking Monitoring System for Pregnant Woman. Proceedings of the 2024 IEEE International Conference on Contemporary Computing and Communications (InC4).

[6] Song K., Zeng X., De Jonckheere J., Koehl L., Yuan X. (2024). An Intelligent Garment for Online Fetal Well-Being Monitoring. Expert Syst. Appl..

[7] Tabassum T., Podder S., Rafid S.T.S. A Comprehensive Framework for Wearable Module for Prenatal Health Monitoring and Risk Detection. Proceedings of the 2024 IEEE International Conference for Women in Innovation, Technology & Entrepreneurship (ICWITE).

[8] Monika S., Battana H., Sangeetha M., Shaik M., Muthusamy J. Analysis of Maternity and Child Health Care System Integrated with IoT and ML. Proceedings of the 2024 IEEE International Conference on Advanced Computing and Communication Systems (ICACCS).

[9] Nalini R., Padmapriyan N., Prabakaran S., Jayanthi K.B. FemmeVibe: Device to Amplify Women’s Health and Harmony. Proceedings of the 2024 2nd International Conference on Artificial Intelligence and Machine Learning Applications (AIMLA)—Theme: Healthcare and Internet of Things.

[10] Altini M., Mullan P., Rooijakkers M., Gradl S., Penders J., Geusens N., Grieten L., Eskofier B. Detection of Fetal Kicks Using Body-Worn Accelerometers during Pregnancy: Trade-Offs between Sensors Number and Positioning. Proceedings of the 2016 Annual International Conference of the IEEE Engineering in Medicine and Biology Society (EMBC).

[11] Altini M., Rossetti E., Rooijakkers M., Penders J., Lanssens D., Grieten L., Gyselaers W. Variable-Length Accelerometer Features and Electromyography to Improve Accuracy of Fetal Kicks Detection during Pregnancy Using a Single Wearable Device. Proceedings of the 2017 IEEE EMBS International Conference on Biomedical & Health Informatics (BHI).

[12] Xu J., Wang X., Wang J., Lin Y., Liu J., Zhang Y., Zhao Y., Chen X. (2022). Fetal Movement Detection by Wearable Accelerometer Duo Based on Machine Learning. IEEE Sens. J..

[13] Ghosh A.K., Catelli D.S., Wilson S., Nowlan N.C., Vaidyanathan R. (2024). Multi-Modal Detection of Fetal Movements Using a Wearable Monitor. Inf. Fusion.

[14] Ghosh A., Shahid O.-I., Nowlan N., Vaidyanathan R. (2024). Comparative Performance Evaluation of Fetal Movement-Detecting Wearable Sensors Using a Body-Worn Device. IEEE Sens. J..

[15] Delay U.H., Nawarathne B.M.T.M., Dissanayake D.W.S.V.B., Ekanayake M.P.B., Godaliyadda G.M.R.I., Wijayakulasooriya J.V., Rathnayake R.M.C.J. Non-Invasive Wearable Device for Fetal Movement Detection. Proceedings of the 2020 IEEE 15th International Conference on Industrial and Information Systems (ICIIS).

[16] Ouypornkochagorn T., Dankul W., Ratanasathien L. (2023). Fetal Movement Detection with a Wearable Acoustic Device. IEEE Sens. J..

[17] Senanayaka J.B., Somathilake E., Delay U., Gunarathne S., Godaliyadda R., Ekanayake P., Wijayakulasooriya J., Rathnayake C. Fetal Movement Identification from Multi-Accelerometer Measurements Using Recurrent Neural Networks. Proceedings of the 2021 IEEE 16th International Conference on Industrial and Information Systems (ICIIS).

[18] Somathilake E., Senanayaka J.B., Delay U., Gunarathne S., Nawarathne T., Withanage T., Godaliyadda R., Ekanayake P., Wijayakulasooriya J., Rathnayake C. Fetal Movement Detection Using Long Short-Term Memory Network. Proceedings of the 2021 10th International Conference on Information and Automation for Sustainability (ICIAfS).

[19] Somathilake E., Delay U.H., Senanayaka J.B., Gunarathne S.L., Godaliyadda R.I., Ekanayake M.P., Wijayakulasooriya J., Rathnayake C. (2022). Assessment of Fetal and Maternal Well-Being during Pregnancy Using Passive Wearable Inertial Sensor. IEEE Trans. Instrum. Meas..

[20] Thilakasiri L.B.I.P., Senanayaka J.B., Gunarathne S., Delay U., Godaliyadda R.I., Wijayakulasooriya J., Rathnayake C. Fetal Movement Identification Using Spectrograms with Attention Aided Models and Identifying a Set of Correlating Parameters with Gestational Age. Proceedings of the 2023 IEEE 17th International Conference on Industrial and Information Systems (ICIIS).

[21] Delay U., Nawarathne T., Dissanayake S., Gunarathne S., Withanage T., Godaliyadda R., Rathnayake C., Ekanayake P., Wijayakulasooriya J. (2021). Novel Non-Invasive In-House Fabricated Wearable System with a Hybrid Algorithm for Fetal Movement Recognition. PLoS ONE.

[22] Rattanasak A., Jumphoo T., Pathonsuwan W., Kokkhunthod K., Orkweha K., Phapatanaburi K., Tongdee P., Nimkuntod P., Uthansakul M., Uthansakul P. (2025). An IoT-Enabled Wearable Device for Fetal Movement Detection Using Accelerometer and Gyroscope Sensors. Sensors.

[23] Ouypornkochagorn T., Ratanasathien L., Dankul W. (2025). A Portable Acoustic System for Fetal Movement Detection at Home. IEEE Sens. J..

[24] TDK MPU-6050 Datasheet. https://www.alldatasheet.com/datasheet-pdf/view/1132807/TDK/MPU-6050.html.

[25] Espressif Systems ESP32-C6 Datasheet. https://www.espressif.com/sites/default/files/documentation/esp32-c6_datasheet_en.pdf.

[26] Choi J.S., Lee J.K. (2023). Effects of Data Augmentation on the Nine-Axis IMU-Based Orientation Estimation Accuracy of a Recurrent Neural Network. Sensors.

[27] Zhou Q., Sun B. (2024). Adaptive K-Means Clustering Based Under-Sampling Methods to Solve the Class Imbalance Problem. Data Inf. Manag..

[28] Xiang G., Miao J., Cui L., Hu X. (2022). Intelligent Fault Diagnosis for Inertial Measurement Unit through Deep Residual Convolutional Neural Network and Short-Time Fourier Transform. Machines.

[29] Spicher L., Bell C., Sienko K.H., Huan X. (2025). Comparative Analysis of Machine Learning Approaches for Fetal Movement Detection with Linear Acceleration and Angular Rate Signals. Sensors.

[30] Hinton G.E., Vinyals O., Dean J. (2015). Distilling the Knowledge in a Neural Network. arXiv.

[31] Liu Z., Mao H., Wu C.-Y., Feichtenhofer C., Darrell T., Xie S. A ConvNet for the 2020s. Proceedings of the 2022 IEEE/CVF Conference on Computer Vision and Pattern Recognition (CVPR).

[32] He K., Zhang X., Ren S., Sun J. Deep Residual Learning for Image Recognition. Proceedings of the 2016 IEEE Conference on Computer Vision and Pattern Recognition (CVPR).

[33] Tan M., Le Q.V. (2019). EfficientNet: Rethinking Model Scaling for Convolutional Neural Networks. arXiv.

[34] Sandler M., Howard A.G., Zhu M., Zhmoginov A., Chen L.-C. MobileNetV2: Inverted Residuals and Linear Bottlenecks. Proceedings of the 2018 IEEE/CVF Conference on Computer Vision and Pattern Recognition (CVPR).

[35] Zhang X., Zhou X., Lin M., Sun J. ShuffleNet: An Extremely Efficient Convolutional Neural Network for Mobile Devices. Proceedings of the 2018 IEEE/CVF Conference on Computer Vision and Pattern Recognition (CVPR).

[36] Tsivgoulis M., Papastergiou T., Megalooikonomou V. (2022). An Improved SqueezeNet Model for the Diagnosis of Lung Cancer in CT Scans. Mach. Learn. Appl..

[37] Wang Y., Yang T., Liang X., Wang G., Lu H., Zhe X., Li Y., Li W. (2024). Art and Science of Quantizing Large-Scale Models: A Comprehensive Overview. arXiv.

[38] Wu H., Judd P., Zhang X., Isaev M., Micikevicius P. (2020). Integer Quantization for Deep Learning Inference: Principles and Empirical Evaluation. arXiv.

[39] Guo H., Li Y., Shang J., Gu M., Huang Y., Gong B. (2017). Learning from class-imbalanced data: Review of methods and applications. Expert Syst. Appl..

[40] Pan Q., Liu K., Zheng S., Wang G. (2025). A Fine-Grained Image Classification Method Based on ConvNeXt Heatmap Localization and Contrastive Learning. IEEE Access.

[41] Griot M., Hemptinne C., Vanderdonckt J., Yuksel D. (2025). Large Language Models Lack Essential Metacognition for Reliable Medical Reasoning. Nat. Commun..

[42] Gou J., Yu B., Maybank S.J., Peng H. (2021). Knowledge Distillation: A Survey. Int. J. Comput. Vis..

[43] Mahajan A., Bhat A. A Survey on Application of Knowledge Distillation in Healthcare Domain. Proceedings of the 2023 7th International Conference on Intelligent Computing and Control Systems (ICICCS).

[44] Gonçalves P.H.N., Bragança H., Souto E. (2024). Efficient Human Activity Recognition on Wearable Devices Using Knowledge Distillation Techniques. Electronics.

